# Barriers and facilitators of healthcare professionals in integrating shared decision‐making in pancreatic cancer treatment: A network approach

**DOI:** 10.1002/cam4.70218

**Published:** 2024-10-14

**Authors:** Joannes Franciscus Adrianus Gerardus van Broekhoven, Fleur Arda Servasa van Heesch, Sasja Mulder, Rosella Hermens, Philip van der Wees, Marion van der Kolk

**Affiliations:** ^1^ Radboudumc, IQ Health Nijmegen Netherlands; ^2^ Radboudumc, Oncology Nijmegen Netherlands; ^3^ Radboudumc, Surgery Nijmegen Netherlands

**Keywords:** Comprehensive Cancer Network, Healthcare Professional Training, Pancreatic Cancer Treatment, Patient Empowerment, Shared decision making (SDM)

## Abstract

**Purpose:**

Shared decision‐making (SDM) is crucial in pancreatic cancer treatment due to its choice‐sensitive nature and limited prognosis. Treatment of pancreatic cancer is organized in a network approach. Several obstacles exist on different levels—patient, healthcare professional, organizational, societal—that impede integration of SDM. This study aims to identify barriers and facilitators to SDM implementation within a comprehensive cancer network.

**Methods:**

A qualitative research design was applied, involving interviews and focus groups on barriers and facilitators with healthcare professionals involved in the implementation of SDM. In one comprehensive cancer network in the Netherlands, including seven hospitals, a project was initiated with the goal of empowering patients and healthcare professionals in SDM throughout primary, secondary and tertiary healthcare settings. A total of 17 participants were assessed. Directed qualitative content analysis was performed by two researchers.

**Results:**

Main findings revealed barriers such as time constraints, lack of priority of physicians, little involvement of general practitioners, and insufficient social context of patients in referrals, alongside facilitators including learning communities with practical SDM examples, metro mapping, involvement of case manager in implementation and patient empowerment strategies.

**Conclusion:**

Addressing cultural, systemic barriers and developing innovative strategies are of importance to enhance SDM in pancreatic cancer treatment in a network approach. This study provides understanding of SDM implementation in complex healthcare settings and offers valuable guidance for future interventions seeking to improve decision‐making processes in pancreatic cancer treatment and beyond.

## 1 INTRODUCTION

Enhancing shared decision‐making (SDM) in the treatment of pancreatic cancer comes with many barriers to overcome. Numerous studies point out the advantages of SDM, but meanwhile note challenges for successful implementation.^1^ The limited prognosis, even with intensive treatment, and high risk for complications make pancreatic cancer treatment a choice‐sensitive treatment, and thus very relevant for SDM.^2^ The multidisciplinary context makes it important to support SDM across the care pathway in the clinical network.^3^

Pancreatic adenocarcinoma is an aggressive form of cancer and chance on curation is only possible in a minority of patients on resectable stages of pancreatic cancer. Consequently, for the majority of patients only palliative treatment is available which involves chemotherapy or best supportive care.^4^ With regard to palliative care, the SDM model with Oncologists and Palliative care specialists (SOP model) has proven to increase acceptance and more conservative treatment decisions.^5^ Meanwhile, in the case of patients with curative intent, pancreaticoduodenectomy and (neo)adjuvant chemotherapy is associated with high morbidity, and substantial mortality.^6^ In almost half of oncological treatment choices, option talk has not been part of the decision‐making process.^7^ Assumptions of power imbalance, both by healthcare professionals as patients can be the underlying cause of this observation.^8^ Additionally, in society the stigma lives that when people are affected by cancer, they have to “fight” against the illness and hold on to every opportunity they get.^9^ This is stimulated with optimism and beliefs of loved ones that patients need support to undergo severe treatment.^10^

Several obstacles exist on different levels—patient, healthcare professional, organizational, and system level—that impede successful utilization of SDM.^11^, ^12^, ^13^ Patients lack knowledge of complications of treating rare diseases^14^ and are unfamiliar with SDM. Healthcare professionals believe that they lack time, sufficient skills and tools to incorporate SDM. The above mentioned patient and healthcare professional factors lead to power imbalance for patients. Likewise, on system level strict guidelines, volume standards and limited resources hinder SDM.^11^ It is important to identify such barriers and develop strategies for implementation of SDM in the treatment of pancreatic cancer across disciplines and organizations, tailored to the barriers found.

Additionally, the multiorganizational comprehensive cancer network, and multidisciplinary context of the diagnosis and treatment of pancreatic cancer may result in lower continuity of care. Continuity of care is related to timely communication, referrals and treatment.^15^ Consequently, this could lead to insufficient coherence of diagnosis and treatment by healthcare professionals and information provided throughout the patient journey.^16^ Referrals to expert centers come with certain expectations for treatment,^11^ and limited coordination and interdisciplinary collaboration hinder shared decision making.^16^ The network approach in this matter is essential to improve continuity of care, provide coherent information and provide seamless shared decision making.

Possible tailored strategies to overcome barriers in the implementation of SDM include obtaining skills of healthcare professionals and using tools, encouragement of patients, and wide investment of the clinical network.^11^ Strategies should include team‐based learning and collaboration between all stakeholders through an integrated network approach.^11^, ^16^ Building forward on these insights the implementation of SDM in the treatment of a rare cancer, such as pancreatic cancer, can be a blueprint for other diseases.

The aim of this study is to identify barriers and facilitators to SDM implementation in pancreatic cancer treatment among healthcare professionals through an integrated network approach.

## 2 METHODS

### 2.1 Design

A directed content qualitative research design was utilized, including individual interviews among healthcare professionals, and a focus group among healthcare professionals, who were part of the development and implementation team of a regional project in the Netherlands to enhance SDM in the treatment of pancreatic cancer in a comprehensive cancer network; Empower2Decide.^17^ The goal of the individual interviews was to obtain in‐depth experiences of healthcare professionals on SDM and its implementation, in a network approach. The goal of the focus group was to reflect on the experiences of the project members regarding factors that had impeded or facilitated network integration of SDM. This paper is prepared in conformity with COREQ guidelines to ensure accuracy.^18^

### 2.2 Setting

**FIGURE 1:**
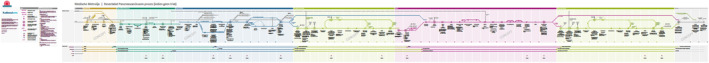
Metro map for pancreatic cancer trajectory. Colors indicate different phases in the patient journey. Due to the elaborated metro map the image has been cut in multiple sections for readability. Colors, from left to right, indicate the following; orange involves diagnostics by the general practitioner, dark green involves diagnostics in the general hospital by the gastro‐enterologist, blue involves diagnostics in the expert center, light green involves neoadjuvant chemotherapy, purple involves the surgical treatment, light green involves adjuvant chemotherapy, gray involves follow‐up by the surgeon or the medical oncologist.

Our qualitative study was conducted within the scope of the Empower2Decide project. In the Netherlands, diagnosis and treatment of pancreatic cancer is organized in eight comprehensive cancer networks.^19^ Recent health service delivery developments increasingly focus on appropriate care by means of SDM in oncological treatment.^20^ Therefore, in one of the eight comprehensive cancer networks, specifically in the region of eastern Netherlands, the Empower2Decide project was initiated with the goal of empowering patients and healthcare professionals in SDM throughout the care pathway across primary, secondary and tertiary healthcare settings. Metro Mapping is a service design tool to represent the complexity of a Care Pathway and can help to optimize, change or redesign the patient journey. It also represents the multidisciplinary collaboration and roles of healthcare professionals. It maps the patient journey across diagnostics, treatment, and follow‐up phases, with options to bypass certain phases, such as choosing chemotherapy over surgery. Also at every phase there is an option to take the path to the general practitioner for solely symptom directed treatment,^21^ see Figure 1. This aligns views of care in critical diagnostics, surgical, oncological, or best supportive care in the case of pancreatic cancer.^22^ In the context of the project, the network developed collaboration and learning communities comprising a total of 80 healthcare professionals, including surgeons, gastroenterologists, oncologists, radiation therapists, casemanagers, nurse specialists, quality advisors, and patients. Implementation of SDM was enhanced by tools (see Table 1) that focus mainly on patients, healthcare professionals and across organizational collaboration.

**TABLE 1:** Tools to enhance SDM within the Empower2Decide network.

Level of influence	Interventions	Involves
Patients	Online patient information	Online website with interactive information on pancreatic cancer, diagnosis, prognosis and treatment options
	“Patient voice” video's	Patients elaborating on their journey and choice
	Promotion to participate in SDM	Video's and campaign information on SDM utilized by whole region
	Audio‐recording of consultation	The service provided by the consulting surgeon to save consultation records in the patient portal
Healthcare professionals	Learning community sessions	Six learning community sessions facilitating peer learning in small groups, discussing best practices
	E‐learning	E‐learning as preparation of learning community session which involves practice of SDM
	Online information on treatment options	Accessible for all healthcare professionals, an online overview of staging criteria, treatment options, process information for referring to multidisciplinary meetings, and literature
Organizations	Promoting use of app based telecommunications	Use of Sillo app enabling timely and easy contact between healthcare professionals of all organizations
	Metromap	Visualization of the patient journey from the general practitioner to diagnostics at the gastroenterologist, specifying therapy routes by surgeons, oncologists, and radiation therapy. This involves consensus on available options at each stage, with designated healthcare professionals responsible for each part of the trajectory
	Fast correspondence	Shortly after consultations and multidisciplinary meetings, updating referring physicians and general practitioners, including clear contact information of casemanagers and involved physicians

### 2.3 Study participants

Study participants for individual interviews were selected based on purposive sampling with the goal to maximize variety of different views and profiles.^23^ The aim was to gather a diverse sample of participants based on three considerations. Firstly, at least from each hospital a healthcare professional was selected. Secondly, because medical oncologists play an important role in the network for the care for patients with pancreatic cancer, more medical oncologists were selected. Hence, medical oncologists also work in the tertiary referring center and are involved in the treatment during the whole treatment trajectory. Thirdly, multiple case managers were selected who serve as primary contact persons for patients and referring physicians, and are involved in the logistics of the treatment. Both professionals extensively listen to experiences of patients with regard to their treatment, and are known with the logistics that are required in this network approach. The study team established a number of 13 interviews beforehand, balancing the expected data saturation and feasibility. The invited sample consisted of 13 healthcare professionals; four medical oncologists, four case managers, two gastroenterologists, a general practitioner, a nurse specialist, and a surgeon. The selection for the different healthcare professionals, including the four medical oncologists, was based on diversity in experience, age, gender, and involvement in the project. The researcher sent information and request for an interview via e‐mail. After 2 week, a first reminder was sent. In case of no response after the second reminder via e‐mail, the eligible participants were called by telephone by the researcher. When they accepted participating, they signed an informed consent form.

Besides the individual interviews with healthcare professionals of the participating hospitals, a focus group was held with project members of the Empower2Decide project. All of the 11 project members who were involved in the implementation of SDM were invited by e‐mail. The invited selection for the focus group included a surgeon, two general practitioners, two medical oncologists, a gastroenterologist, a patient representative, three policy officers and implementation experts, and a communications expert. In case of no response, a reminder was sent via e‐mail after 1 week, and in case of no response, they were called by telephone. When project members participated in the focus group, they signed an informed consent form.

### 2.4 Data collection

In‐depth semi‐structured individual interviews were held using a topic list. The topic list was derived from earlier survey results on collaboration,^24^ components of the project, and relevant literature. Key components of the topic list were experiences with SDM in the treatment of pancreatic cancer, opportunities to apply SDM and experiences with tools for SDM, provided during the Empower2Decide project (Appendix B). Data analysis and data collection went in an iterative approach, in which missing information of former interviews were delved into in consequent interviews. Interviews were conducted one‐on‐one via video‐conference in Microsoft Teams and were audio recorded. Interviews occurred in April and May 2022. JvB and FvH executed interviews, see Appendix A.

For the focus group, an extensive topic list was made as well, based on literature and previous surveys in the network with regard to implementation and collaboration. This list was condensed for use in the focus group (Appendix C). The focus group was organized face‐to‐face and took place in May 2022.

The interviews and focus group were audio‐recorded and transcribed verbatim. Transcripts were not returned for comments unless specifically requested.

### 2.5 Data analysis

Directed qualitative content analysis was applied based on specific theory based intervention components.^25^ Coding and analysis was carried out using Atlas.ti version 9.1.6 coding software (JvB and FvH). Assarroudi's^25^ method provides a 16‐step guide to perform manifest content analysis of both the interviews' and focus group transcripts. In a formative organization matrix categories were formulated, based on theory along with potential new categories, which emerged in an inductive approach (JvB and FvH). The main categories were based on the Consolidated Framework for Implementation Research (CFIR).^26^ The CFIR is a comprehensive framework designed to systematically identify and organize barriers and facilitators that influence the implementation process. It encompasses five key domains: innovation characteristics, inner setting, outer setting, individuals involved, and the implementation process. Simultaneously, inductive coding allowed for the exploration of emergent patterns, which were further developed through axial coding of transcripts (JvB and FvH). The directed qualitative content approach ensured structure of analysis, while enabling depth, capturing both predefined and novel insights. Eventually, codes were categorized to main categories and subcategories. To ensure credibility, coding was conducted independently by JvB and FvH and discordances were discussed until consensus was reached. Axial codes were verified in the entire research team.

### 2.6 Reflexivity statement

Related to the persons involved in this research, as main interviewing researchers, JvB and FvH, recognize that their professional backgrounds and personal experiences in healthcare may have influenced their approach to this study. JvB is practicing as MD as surgery resident and FvH is in training for MD. MvdK is chair of the division of gastrointestinal and oncologic Surgery, specialized in pancreatic surgery and developer of complex ICU related Care pathways at Radboudumc. She is initiator and leader of the Empower2Decide project. SM is medical oncologist and is involved in innovation in healthcare and involved in the lead of Empower2Decide. PvdW and RH are professors at a science department of the University hospital and are respectively involved in value based healthcare and personalized oncological care. This background likely shaped their interpretation of the data, especially in recognizing the importance of collaboration and the challenges associated with implementing SDM in oncological care networks. Throughout the research process, JvB and FvH were conscious of the professional connection between themselves and the study participants, facilitating openness and understanding during interviews and the focus group, potentially leading to richer thoughts. However, this may have introduced some bias, as participants might have felt a sense of collegiality that influenced their responses. Despite this, the junior status of JvB and FvH compared to the more experienced interviewees likely minimized this effect.

## 3 RESULTS

Results of the individual interviews and the focus group are presented collectively. Ten out of thirteen invited healthcare professionals were individually interviewed to gain an in‐depth understanding of experiences regarding the network‐approach to enhance the implementation of SDM (Table 2). Two medical oncologists and one gastroenterologist declined to participate in the interviews due to time constraints, despite multiple reminders. The duration of interviews was between 22 and 64 min (mean 39 min) and important components were views on SDM, collaboration and components of the project.

**TABLE 2:** Functions, hospitals, and respondent codes of interviewees.

Function	Hospital	Respondent code
Nurse specialist	A	R1
Casemanagers	B	R2
	C	R4, R5
	A	R7
Surgeon	A	R3
General practitioner	D^a^	R6
Medical oncologists	A	R8
	B	R10
Gastro‐enterologist	C	R9

^a^ General practitioners work at independent primary care practices. They mostly collaborate with and refer to the nearest general hospital.

Seven out of the eleven project members participated in the focus group consisting of a surgeon, gastroenterologist, general practitioner, two policy advisors and a patient representative (Table 3). One general practitioner, one medical oncologist, one policy advisor, and one communications expert from the project group were unable to participate due to lack of time. The patient representative deceased in 2022 during the end of the project. The duration of the focus group was 2 h and 4 min and involved mainly facilitators and barriers of implementation of network integration of SDM.

**TABLE 3:** Functions, hospitals, and respondent codes of focus group participants.

Function	Hospital	Respondent code
Surgeon	A	R11
Policy officers	A	R12, R17
General practitioner	C^a^	R13
Medical oncologist	A	R14
Patient representative	–	R15
General practitioner	D	R16

^a^ General practitioners work at independent primary care practices. They mostly collaborate with and refer to the nearest general hospital.

Experiences of healthcare professionals and project members with the multi‐organizational implementation of SDM are organized around identified barriers and facilitators in each of the CFIR domains (see Table 4). In order of the CFIR domains the result are presented.

**TABLE 4:** Barriers and facilitators within multi‐organizational implementation of SDM.

Facilitators	Barriers
Innovation	
Metro mapping as tool	Lack of practical examples of SDM
Online patient information	
Learning communities with patient representatives	
Addressing participation in SDM	
Inner setting	
Sillo, app based telecommunications, to discuss logistic matters (interorganizational)	Lack of complete transfer of patient information and social contexts
Outer setting	
	Lack of care chain financing for oncological care
	Lack of attention to SDM in guidelines or education
Individuals involved	
Involving casemanagers in the implementation	Insufficient involvement of general practitioners
	Insufficient dedicated time or other priorities of involved healthcare professionals
	Rigid to change and implement enhanced SDM
Process	
Physical meetings to meet in person	
Online meeting to reduce travel time of healthcare in more remote regions	
Word of mouth; telling others about the project	

### 3.1 Innovation

Implementation tools used in the promotion of SDM in the patient journey include metro mapping, providing patient information, learning communities, and education and training in SDM (see Table 1). The interviews revealed several barriers and facilitators for using the tools. Participants generally experienced that the project was effective in fostering collaboration across organizations and supporting SDM throughout the patient journey.

*Metro mapping* is experienced by participants as a facilitating implementation characteristic with co‐created steps in the patient journey, and directing which physician is responsible for the patient at what moment in the journey. Consequently, participating healthcare professionals were aware of the options available and could incorporate this in consultations with patients. Most importantly, participating healthcare professionals valued clarifying the care path using the service tool metro mapping and providing information to patients via a regional website. Defining the care pathway by composing the medical metro map was considered a facilitator to enhance SDM. Particularly defining all decision moments with patients and making clear what information should be communicated by whom was considered very useful.

What I thought was very strong is that the diagnostic aspect the patient goes through is defined better and we are more aware of that, within the network. We realize what our role is and know what happens in other places. It has given me more awareness about shared decision‐making and being more aware of setting up a conversation that way.—R14.

I think it is good that it is harmonized a bit more in that regard between the different caregivers involved with a patient, so patients hear a little more of the same sound or nuance in it.—R3.

I really liked the metro map for that. We have used this to map out the entire process between all lines of care: for regional hospitals, academic hospitals and GPs. All decision moments are included. This ensures that you are more aware of the process you are facing.—R9.

A second facilitator for implementing SDM was empowering patients to obtain information on their options and the process of SDM. The patient was more informed about important decision moments, which enabled better preparation. Informed patients were seen to ask more concrete questions, contributing to a well‐considered decision. The regional *online patient information* offered an overview of the required information and included patients' experiences, which others might recognize. Additionally, the neutral design of the website made it applicable for collaborating hospitals.

That patients already know about shared decisions and that an important decision moment is coming. That is why we talk about the pros and cons of different treatment options.—R1.

Another specific facilitator mentioned by participants was *patient participation in learning communities*, which enhanced insight into views of SDM. This created awareness of SDM by helping healthcare professionals comprehend how patients experienced the decision‐making process. Furthermore, an important lesson entailed *introducing SDM at the start* of a consultation and stating what will be discussed. This ensured clarity and that the patient was prepared for what was to come.

It is not something you can apply right away, but it creates self‐awareness. You realize how the patient experiences the process.—R4.

However, in the learning communities, healthcare professionals preferred to *receive practical examples of how SDM* is best applied. The lack thereof was see as a barrier, and they would like to see examples of a good SDM process.

I would focus more on practicalities because at this moment we keep hanging in shared decision‐making, but I would like to see more practicality: how can you create conditions in such a way that you can manage it with multiple hospitals? And that is a well‐developed care path, good patient communication, and unambiguous patient communication. That would make me happy.—R2.

### 3.2 Inner setting

The inner setting involved former collaboration, communications and digital information transfers between gastroenterologists, surgeons, and medical oncologists in the region. The interviews unveiled various obstacles and enablers for the inner setting.

*Lacking complete transfer of patient information* in patient referrals could miss patients' social contexts. Consequently, information of personal values of patients could possibly be not addressed, and may result in worse SDM.

Personal *interprofessional communication* via *Sillo*, a secure medical messaging app, facilitated SDM in the sense that personal patient information could be shared. The interviewed medical specialists discussed via the app about, for example, expert panels, experimental studies, questions about follow‐up treatments, or a potential referral, but not so much about medical matters. This simplified easy contact on logistical matters of patient treatment.

Colleagues contact me to ask if there is a study for someone. Or if we have a joint patient, a question about follow‐up treatment or whether or not they should refer someone.—R8.

I use Sillo to contact, can we call or would you like to discuss that patient for me in the multidisciplinary tumor board.—R10.

### 3.3 Outer setting

Outer setting aspects mentioned by the participants included lacking financial incentives for the organization of oncological regional care and the limited presence of SDM in medical education and guidelines. Specific barriers were the *lack of care chain financing for oncological care* and the administrative burden of oncological care. Due to these two factors, it was hard to coordinate overlapping tasks with each other. There was also a lack of a proper financing system for SDM across multiple organizations.

Overlapping tasks cannot be coordinated easily because the financing is so complicated, and there is no chain financing. There is a huge workload in the surgery department with a lot of improper administrative tasks. (…) If I want to see a patient, orders have to be made by the casemanagers.—R8.

Another barrier in the outer setting was the *lack of attention to SDM in guidelines* or the education of older healthcare professionals; guidelines were strict and did not stimulate SDM.

I think that shared decision‐making could be paid a little more attention to within my discipline.—R1.

Cancer and shared decision‐making are complicated. In the education for GPs, nothing is included on cancer and shared decision‐making. I always say this is because no guidelines or standards are available. There is no material to build on, too little material. (…) There is material, but it is not as readily available as guidelines and standards that are simply there.—R13.

### 3.4 Individuals involved

Individuals involved in the implementation ensured the collaboration of all disciplines and involved hospitals. A prominent facilitator shared by interviewed physicians, casemanagers, and nurse specialists was *involving casemanagers in the implementation* of SDM, since often casemanagers of different hospitals would not be in contact. Due to the project, protocols and best practices were shared to enhance a complete overview by sharing all patient‐related information.

I enjoyed networking with each other and having insight into each other's work because you see that the role of the casemanager is fulfilled very differently in every hospital and I believe there is a lot to achieve with each other.—R2.

A barrier mentioned by participants was the *insufficient involvement of GPs*, especially in tertiary care. By limited reaching out to the GPs in the decision period, an important facilitator for collaboration was missed. GPs could provide more guidance to patients with a critical illness.

GPs often consciously choose to take a step back in case of a severe diagnosis. They leave it to the hospital and come back into the picture when it is over. I think there could be a little more guidance in that. In return, a hospital may also need better communication to the GP.—R2.

In my opinion, the GP is too little involved in this.—R3.

Perceived barriers to participating in all implementation and learning activities for healthcare professionals were a *lack of time or having other priorities*. Additionally, this lack of time also posed a risk of falling back into old habits, thus acting as a barrier to sustainable implementation.

I have not been a very active participant in the project, simply due to a lack of time.—R10.

I was even supposed to be a process counselor for part of the learning community last week, but at the last minute another appointment came up that I had to give priority. That is how it goes.—R3.

To suddenly perform a consult in a completely different way, you have to do it consciously for a long time. After that, it will be automatic. Often, especially in recent years due to the business, it is just a part of the day, I think. That is much more difficult.—R6.

Another perceived barrier by respondents was that some healthcare professionals were thought to be rigid for *not being open to change* and, therefore, not willing to participate in a project implementing SDM.

I think that the specialists should join the project, but they are a bit rigid about it. (…) They are not open to change.—R4.

### 3.5 Process

Process factors included the frequency of meetings and the hybrid format of meetings due to the COVID‐19 pandemic. Learning communities were organized both face‐to‐face and online. Primarily, physical meetings were held with restrictions. However, due to more experiences and potential benefits of online meetings, more meetings were held online. The last two meetings were organized face‐to‐face for a practical training and presentation of results. The variation of sometime physical and online meetings ensured both advantages. Some participants indicated that *physical meetings were preferable* because of the possibility of meeting with each other and matching the faces of the people with voices they often had telephone calls with. This enabled collaboration. However, an argument in *favor of online meetings was no need for to travel* for participants from afar, for whom travel time could be 2 h. These participants were able to take part more often online.

I think a physical meeting is much more rewarding for me than online meetings. But there was something of a virus. It was not always possible. On the contrary, online takes much less time for colleagues in other hospitals. They might consider the online possibility as a gain. But like this more than online.—R1.

I prefer physical meetings because you meet your colleagues. You will create more cooperation with that. Then you hear more about how they work in other hospitals.R4, R5.

It certainly has advantages, we have been able to do a lot of meetings very efficiently and we would never have been able to do that if we had to get everyone together live.—R17.

However, participants mentioned that the COVID‐19 pandemic did bring opportunities. Namely, during the COVID‐19 pandemic, the project group composed a medical metro map of the COVID‐19 care path. This showed that the methodology of the metro map was applicable to other care settings. It also provided branding for the development of medical metro maps.

The description of the care path (development of the COVID‐19 metro map) has been carried out by the project group members (…) Although it concerns different types of care and different healthcare professionals, the working method and methodology of shared decision‐making and the use of the metro map are universally applicable. In that sense, Empower2Decide benefits substantively from this development, and it has an important PR‐function for shared decision‐making. The use of the COVID‐19 metro map responds to current events and can serve as a flywheel for the implementation of shared decision‐making in other oncological and non‐oncological care paths.—R13.

A facilitating factor was *word of mouth*; telling others about the project. This contributed to more awareness about SDM and its importance. It was a sign that the implementation was proceeding.

It is especially nice to see people are announcing it and that is an expression of the actual implementation.R12.

## 4 DISCUSSION

The study identified system and individual‐level barriers and facilitators for the network approach for SDM. Barriers include strict guidelines, a lack of financial incentives for collaboration, limited information transfer, and the relatively low priority given by some healthcare professionals, all of which restrict the effective integration of high‐level SDM in the context of pancreatic cancer treatment within a regional network. Facilitators are the use of learning communities with practical skills in SDM, metro mapping, and involvement of casemanagers.

### 4.1 Comparison with previous literature

Several studies have been conducted in terms of multiorganizational involvement and stepwise implementation of SDM, the MAGIC study in the NHS,^11^ the PREPARED trial in Germany,^16^, ^27^ the SDM:HOSP trial in southern Denmark,^28^ have implemented hospital‐wide approaches to SDM. However, specifically focusing on multiorganizational enhancement of SDM in rare care paths, such as pancreatic cancer, with involvement of primary, secondary and tertiary healthcare professionals, is unprecedented. This enables specific attention to a dedicated group of healthcare professionals. In contrast, the rarity of only pancreatic cancer care journey reduces resources and senior‐level buy in, which is emphasized as a facilitator in the previously research.^11^, ^16^ Building forward on overcoming interorganizational barriers, our study depicts a bridge in the form of consensus on decision moments in the patient journey, learning communities to discuss best practices and smooth transfer of information. Consensus is obtained through metro mapping.

Our study pioneers with metro mapping as method for co‐designing care pathways and associated decision moments in the treatment of pancreatic cancer. Today, despite the original development paper,^22^ no empirical research has been published with regard of the metro mapping. Despite internationally care paths are developed, little studies emphasize incorporation of SDM moments in care pathways development.^11^, ^29^

Limited priority of particular senior healthcare professionals within an organization, little incorporation of SDM in guidelines and lack of financial incentives are similar barriers as in previous research.^11^, ^16^, ^30^ Our study did address the barrier of the application of sociological and contextual information of patients in the discussion in multidisciplinary meetings, due to the lack of patients' context in information transfer. Likewise, Scholl et al.^27^ experienced lower discussion of patients' views in the implementation phase, which could be explained by lacking transferability of electronic health records.

Presenting this paper, a blueprint for interorganizational collaboration to implement SDM had been proposed for patients with a rare type of cancer, which involves many disciplines and organizations, such as pancreatic cancer. Other patient groups could be selected for application of this blueprint and the identified barriers and facilitators could be verified.

### 4.2 Strengths and Limitations

A strength in this, guided by a CFIR as theoretical framework, the method provides structure, theoretical rigor and enhances comparability with other evaluations of implementation of SDM studies.^27^ Secondly, our findings are set in the “real world” regular care for patients with pancreatic cancer and thus the ecological validity is high. Thirdly, building forward on the content analysis approach, including directed qualitative content analysis, enables a detailed examination of the data. The use of Atlas.ti for coding and peer examination enhances the transparency and reproducibility of the analysis.

Although a patient representative in the project group and in the focus group, the current study does not address the patient perspective in the implementation of SDM in the network approach. Since patients experience the pancreatic cancer journey only once, they cannot compare their experience before and after the Empower2Decide intervention. However, further research should investigate patients' experiences with the network approach in pancreatic care to identify any unmet needs.

Despite the decline of interviews with a gastro‐enterologist and two medical oncologists from two hospitals due to their lack of time, saturation of most data was achieved, because in the last interviews most themes were repeated without generating new insights related to the network approach. However, with regard to the role of general practitioners no saturation was achieved with only one general practitioner being interviewed. Nevertheless, a recent study by Abma et al. elucidates that general practitioners experience a limited role in SDM in the treatment of pancreatic cancer, and that general practitioners are mostly involved in a supporting role. To take this role, general practitioners require timely updates from medical specialists including treatment options and contextual details.^31^ Future research is warranted to research the desired role of the general practitioner in the SDM process for pancreatic cancer.

When reflecting on the decline of physicians from two hospitals to participate in the interviews, we observed that those hospitals were less involved in the project and this also resulted in limited participation in this research. This emphasized their limited priority and lack of time as barriers for collaboration and implementation. In addition, these two hospitals are located at the regional borders of our network. Their geographic location may explain why not all physicians were committed to referring patients to the tertiary hospital in our network. In future research specifically the criticaster and the healthcare professionals with less priority for the network approach for implementation of SDM should be selected. Hence, this could identify important barriers for whole network involvement and potentially enhance broad support for the implementation of SDM.

Another limitation of this study is not including palliative care specialists in the collaborative network. This project aimed to implement SDM among patients with curative options, and we did not consider palliative care as scope of our study. However, there were cases where decisions for non‐curative treatment or advanced cancer management were made. In such instances, some patients received palliative chemotherapy from the medical oncologists in our network, while those requiring symptomatic treatment were supported by their general practitioners within their community. Given recent development in shared decision making and palliative care, it is evident that future network approaches should incorporate palliative care specialists into the network approach to implement SDM.^5^

## 5 CONCLUSION

In conclusion, the implementation of SDM in the treatment of pancreatic cancer requires a multifaceted approach that addresses barriers at the patient, healthcare professional, and organizational levels. The Empower2Decide intervention presents a comprehensive network approach to overcome these barriers and enhance SDM. The study provides valuable insights that can inform future interventions aiming to improve decision‐making processes for pancreatic cancer patients and beyond. This study's findings can be applied to the development of care pathways in tertiary hospitals for patients with other cancers to integrate recognizable SDM moments. The results of this study may also be translated to other patient groups with diseases for which intensive treatment options exist and who receive treatment in a network approach. This is especially relevant when collaboration and information sharing between general practitioners, general hospitals, and tertiary hospitals is essential to deliver seamless SDM.

## AUTHOR CONTRIBUTIONS

**Joannes Franciscus Adrianus Gerardus van Broekhoven:** Conceptualization (equal); data curation (lead); formal analysis (lead); investigation (equal); methodology (equal); project administration (lead); resources (equal); software (equal); visualization (lead); writing – original draft (lead); writing – review and editing (lead). **Fleur Arda Servasa van Heesch:** Data curation (equal); formal analysis (equal); methodology (equal); project administration (supporting); writing – original draft (supporting); writing – review and editing (supporting). **Sasja Mulder:** Conceptualization (equal); funding acquisition (equal); methodology (equal); supervision (equal); validation (equal); writing – review and editing (equal). **Rosella Hermens:** Formal analysis (equal); methodology (equal); supervision (equal); validation (equal); writing – review and editing (equal). **Philip van der Wees:** Conceptualization (equal); funding acquisition (equal); investigation (equal); methodology (equal); supervision (equal); validation (equal); writing – review and editing (equal). **Marion van der Kolk:** Conceptualization (equal); funding acquisition (lead); methodology (equal); project administration (equal); supervision (equal); validation (equal); writing – review and editing (equal).

## FUNDING INFORMATION

This study was conducted due to a grant from Zorginstituut Nederland (Transparantie2019 Empower2Decide) for developing a regional cancer network aiming to improve shared decision‐making. Zorginstituut Nederlands is a Dutch Government advisory body. The funding body had no role in the study design, execution, analysis, interpretation of the data or the decision to publish results. The funding body had no role in the writing of this manuscript.

## CONFLICT OF INTEREST STATEMENT

All authors declare they have no conflicts of interest.

## ETHICS STATEMENT

Ethical approval is not required for this type of study under Dutch law, and an exemption was obtained by the local Medical Ethics Committee ‘CMO Regio Arnhem‐Nijmegen’ (file number 2019–6053).

## Data Availability

The data that support the findings of this study are available on request from the corresponding author, JvB. The data are not publicly available due to restrictions for example, their containing information that could compromise the privacy of research participants.

## APPENDIX A Interviewer characteristics and data processing

### A.1

JB is male and FH is female. Both had no existing relationship with the participants and had no assumptions before the study started as this was a new topic to both researchers. JB has followed several interview trainings. FH had followed a course on qualitative research (including interview training) before commencement of the study.

Data processing: All data collected during this study were stored on a secured server of the Radboud University Medical Centre, a tertiary hospital in the eastern Netherlands. Only JB and FH had access to the personal details of the participants. Transcription was conducted partly by FH and partly by a transcription service that is affiliated with Radboud University Medical Center and that ensures privacy. All transcripts were anonymised before being uploaded into Atlas.ti.

## APPENDIX B Interview guide

### B.1

Part 1: General questions.

1. Do you feel you have sufficient knowledge and skills to apply SDM?
2. To what extent is your vision on SDM in line with Empower2Decide?
3. How does the Empower2Decide project contribute to SDM and regional cooperation in the network?
  - What has changed as a result of the project?
  - Is the approach used also useful for other chains/tumor types?
4. What do you think of the cooperation between primary, secondary and tertiary care? (specially ask further questions about collaboration with the general practitioner, casemanager/nursing specialist and physician)
  - What is going well and what could be improved?
5. What has the Empower2Decide project contributed to this? How?

Part 2: Questions per empower2decide implementation activity.

1. Did you participate in the learning community meetings?
  1. If not, why did you never participated in the learning community meetings?
  2. If yes:
    - What did you think of the learning community meeting(s)?
    - How did the learning community meeting(s) benefit you?
    - Why did/did the learning community meeting(s) meet your expectations?
  1. What did you expect differently?
    iv What did you do/apply differently after the learning community meeting(s)?
    v Would you recommend participation in the learning community meeting(s) to colleagues. Why/why not?
    vi What are tips for next learning community meetings?
2. Did you participate in observations in the consulting room?
  1. If not, what is the reason having never participated in the observations in the consulting room?
  2. If yes:
    - What did you think of the observation in the consulting room and the feedback received?
    - How did the observations in the consulting room benefit you?
    - Why did/did the observation in the consulting room meet your expectations?
    - What did you do/apply differently after the observation?
    - Would you recommend participation in the observations in the consulting room to colleagues. Why/why not?
    - What are tips for the observations in the consulting room?
3. Do you use the Siilo application?
  1. If not, what is the reason why are you not using the Siilo app?
  2. If yes:
    - How/what do you use the Siilo application for?
    - What are your experiences with the Siilo application?
    - Would you recommend using the Siilo application to colleagues? Why/why not?
4. Do you refer patients to the regional website?
  1. If not, what is the reason for not doing it?
  2. If yes:
    - How does the regional website benefit you? How does the information on the regional website help you or not in SDM with patients?
    - What response do you get back from patients about the regional website?

(ask to GPs only)

5 How are you involved in SDM of patients with pancreatic cancer?
6 What do you like about that?
7 What do you dislike about that?
8 Which aspects have been improved by the Empower2Decide project?
9 How do you experience mutual communication and cooperation in the network in the process of SDM?
10 Do you have the opportunity to ask questions to the medical specialist if necessary? Would you like to explain this?
11 Do you have the opportunity to provide additional information about a patient to the medical specialist if necessary? Would you like to explain this?
12 There is an NHG guideline (2014) which describes the role of the GP in oncological care. To what extent is this discussed in the GP education or training?

Part 3: Experiences with the empowr2decide project in general.

1. What has changed with regard to SDM within the network compared to before the start of the Empower2Decide project? What has changed with regard to transfer and collaboration?
2. How do you look back on what the project has yielded, compared to the expectations you had beforehand? Does it meet the expectations you had? Why/why not?
3. Looking back on the project, what could/should we have done differently?
4. If the activities were offered to hospitals on a larger scale in the future, what would you like to change? Do you have suggestions for improvement? (ask per activity)

- Learning community meetings
- Observations in the consulting room
- Siilo application
- Development of the regional metro map for patients with pancreatic cancer
- Regional website for both healthcare professionals and patients with the animation ‘samen beslissen’, patients metro map, patient voices, information about treatments, forecasts, healthcare professionals, etc.

5 What future possibilities do you see and with regard to the process of SDM?
6 What can be your role in advancing the implementation of SDM?
7 What wishes do you still have with regard to the SDM process?
8 After the empower2Decide project, what is still needed within your institution to further implement the SDM process and promote regional cooperation?

- What else can we do for you to facilitate SDM with patients with pancreatic cancer? What can we do to facilitate the information transfer of collaboration in the network?

9 What did you think of the support/guidance of the Empower2Decide project team? What did you like and what could be better?
10 Do you have any questions or do you want to add something?

## APPENDIX C Focus group guide

### C.1

Introduction subject and goal focus group.

Appoint the role of chairman and scribe.

Topics.

1. In general, what are your experiences with the implementation of SDM using the E2D program? (to be told spontaneously by the stakeholders).
2. What implementation activity contributed most to success? What implementation activity contributed the least?
3. What were barriers and what were facilitators to the implementation of SDM in a network setting?
4. What is still needed in this region to amplify the effect?
5. Under what conditions can E2D be extended to other networks?

Summary and conclusion.

Closing.
